# Simulation of 3D Electrochemical Phase Formation: Mixed Growth Control

**DOI:** 10.3390/ma14216330

**Published:** 2021-10-23

**Authors:** Vladimir A. Isaev, Olga V. Grishenkova, Alexander V. Kosov, Olga L. Semerikova, Yuriy Zaikov

**Affiliations:** Institute of High Temperature Electrochemistry, Ural Branch of the Russian Academy of Sciences, 620990 Yekaterinburg, Russia; v.isaev@ihte.uran.ru (V.A.I.); Alexander.kosoff@yandex.ru (A.V.K.); o.semerikova@ihte.uran.ru (O.L.S.); Zaikov@ihte.uran.ru (Y.Z.)

**Keywords:** electrocrystallization, kinetics, nucleation, growth

## Abstract

Processes of nucleation and growth largely determine the structure and properties of thin films obtained by electrodeposition on foreign substrates. Theoretical aspects of the initial stages of electrochemical phase formation under constant and variable overpotentials are considered in this work. Simulation of multiple nucleation with mixed (charge transfer, and diffusion) controlled growth was performed for three cases (cyclic voltammetry, potentiostatic electrodeposition, and galvanostatic electrodeposition). The influence of the bulk concentration of depositing ions and the exchange current density at the electrolyte/nucleus interface on cyclic voltammograms (CVs), transients of current and overpotential, as well as the number and size of non-interacting new-phase nuclei was analyzed. It is found that, under galvanostatic conditions, the number of nuclei decreases as the concentration of depositing ions increases due to a more rapid decrease in overpotential. The proposed model was applied to determine the diffusion coefficient, exchange current density, and transfer coefficient considering the experimental CV.

## 1. Introduction

Nucleation and growth of three-dimensional nuclei are typical initial stages of metal deposit formation on the surface of an indifferent electrode during the electrocrystallization. Studying the mechanism and kinetics of these processes is important both for the successful control of the morphology and properties of electrolytic coatings and for the development of concepts regarding the fundamental regularities of electrochemical phase formation [1,2,3].

Certain information on the processes of nucleation and growth can be obtained using various electrochemical techniques; their advantages include the ability to set and control supersaturation (overpotential) and to record accurately the response of the system. Usually, experimental potentiostatic current transients are analyzed within the framework of well-known theoretical models for nucleation with diffusion-controlled growth [3,4,5,6,7,8,9,10]. However, the calculated values of the number density of nuclei, the nucleation rate, and the growth rate can significantly differ from those obtained by the electron microscopy [2,11,12,13,14,15] due to the use of numerous approximations, which consider the mutual influence of nuclei through the overlap of neighboring nuclei or their diffusion zones; other reasons for the discrepancies are discussed in [16]. An exact solution to the overlap problem can be found only for the case of kinetically controlled growth [17,18], which is relatively rarely realized under ordinary conditions of electrodeposition [19,20]. The growth of a new phase can often occur under mixed (charge transfer and diffusion) control [21,22,23,24,25,26]. Taking into account that the overlap significantly complicates the determination of the nucleation and growth parameters in this case, they can be most accurately detected by the analysis of the data on the formation of single nano- and microcrystals or data on the initial stages of growth at multiple nucleation, when the influence of nuclei on each other can be neglected [6,27,28,29,30,31,32,33,34].

Several models have been developed for 3D nucleation followed by mixed growth control using different approaches. Altimari and Pagnanelli [24,25] derived a model for electrochemical nucleation and growth of metal nanoparticles under mixed kinetic-diffusion control using the concept of planar diffusion zones [6] and obtained an approximate analytical expression to compute the potentiostatic current transient. Milchev and Zapryanova [22,23] studied the progressive nucleation and growth of copper crystals on a glassy carbon electrode and analyzed the time dependences of the current and the number of nuclei at constant overpotentials in terms of the proposed theory, which considers a two-stage electrochemical reaction and growth under combined (charge transfer and diffusion) limitations. This model was also applied to study the nucleation and growth kinetics of Pd nanoparticles by analyzing the initial parts of experimental potentiostatic current transients in [26]. Milchev [21] considered the formation and growth of spherical clusters in the case of multi-step electrochemical reactions and direct attachment mechanism and derived theoretical expressions for the time dependences of the linear cluster size, growth current, as well as current–time relationships for progressive and instantaneous nucleation. Abyaneh et al. theoretically investigated the growth of a single hemispherical center under a mixed kinetic-diffusion-controlled mechanism in non-steady-state conditions and the steady-state approximation and determined the rate constant ranges, for which the growth process is controlled by the charge transfer rate, by a mix of charge transfer and diffusion, and only by the diffusion rate [28]. Mamme et al. [33,34] investigated the growth of a single hemispherical silver nucleus using the multi-ion transport and reaction model that considers diffusion and migration of all ions and the change in the nucleus size in accordance with Faraday’s laws. The calculations were performed using a finite element method and the simulated dependences were in good agreement with the experimental curves obtained by two techniques (chronoamperometry and linear sweep voltammetry with rotating disk electrode). Modeling potentiostatic current transients demonstrate that the transition from kinetic to mixed control and then to diffusion control occurs as the nucleus grows, and the transition times depend on the overpotential, concentration, and initial nucleus size [33].

This work is aimed at the theoretical analysis and simulation of the formation and mixed-controlled growth of non-interacting hemispherical nuclei on an indifferent electrode for three basic electrochemical techniques (potentiostatic and galvanostatic electrodeposition, cyclic voltammetry) within the general scheme. The cathodic current and overpotential are considered positive in this work.

## 2. Model and Calculation Method

In this paper, we use the approximations of the classical nucleation theory (CNT), which are valid at moderate supersaturations (overpotentials), when macroscopic parameters can be applied to describe the properties of 3D new-phase nuclei [35,36,37]. The basic CNT equation (the Volmer–Weber equation) has the following form for the electrochemical nucleation:

(1) $$J(t)=K_{1}\exp(-K_{2}/\eta^{2}),$$

where *J* is the nucleation rate, *t* is the time, η is the overpotential, and *K*_1_ and *K*_2_ are nucleation constants. The time dependence of the number of nuclei formed on the electrode with the surface area *s* can be found using:

(2) $$N(t)=sK_{1}\overset{t}{\underset{0}{\int }}\exp(-K_{2}/\eta^{2})d\tau .$$

The radius of the hemispherical nucleus of critical size is described by the Gibbs–Thomson relation,

(3) $$r_{c}=2\sigma \upsilon /ze\eta ,$$

where σ is the surface tension of the electrolyte/nucleus interface, υ is the volume of one new-phase atom, *z* is the valence of depositing ions, and *e* is the elementary electric charge.

If the growth of the supercritical nucleus is controlled both by the charge transfer and by the diffusion of depositing ions in the electrolyte to the nucleus surface, then [36,37]:

(4) $$i_{g}=i_{0}\left[\frac{c_{\mathrm{sr}}}{c_{0}}\exp\;\alpha f(\eta -\eta_{p})-\exp\beta f(\eta_{p}-\eta )\right],$$

where *i*_g_ is the growth current density, *i*_0_ is the exchange current density at the electrolyte/nucleus interface, *c*_sr_ is the concentration of depositing ions near the surface of the growing nucleus, *с*_0_ is the bulk concentration of these ions, α and β are the transfer coefficients (α+β=1), f=ze/kT, *k* is the Boltzmann constant, and *T* is the absolute temperature, and

(5) $$\eta_{p}=2\sigma \upsilon /zer.$$

The term η_p_ (so-called phase overpotential) considers the Gibbs–Thomson effect on the growing nucleus; the *r* radius nucleus exists in unstable equilibrium with the electrolyte at η = η_p_.

Diffusion to small objects can be considered stationary; therefore, the solution of the Fick equation in spherical coordinates for semi-infinite diffusion in the stationary approximation [38,39,40] can be used to determine *c*_sr_:

(6) $$c_{\mathrm{sr}}=c_{0}-i_{g}r/zeD.$$

The general expression for the growth current density of the hemispherical nucleus under mixed control is obtained by combining Equations (4) and (6):

(7) $$i_{g}=\frac{\exp\;\alpha f(\eta -\eta_{p})-\exp\beta f(\eta_{p}-\eta )}{\frac{1}{i_{0}}+\frac{r\exp\;\alpha f(\eta -\eta_{p})}{zec_{0}D}}.$$

The time dependence of the nucleus radius can be found by the formula:

(8) $$\frac{dr}{dt}=\frac{i_{g}\upsilon }{ze}.$$

Equations (7) and (8) provide a complete description of the growth kinetics of the new-phase nucleus on the surface of an indifferent electrode for a given dependence η(*t*). In the case of formation and independent growth of *N* nuclei, these expressions can be supplemented by Equation (2) and

(9) $$I=\underset{N}{\sum }I_{g},$$

where *I* ≡ *I*(*t*) is the current and $I_{g}=2\pi r^{2}i_{g}$. The time dependence of the current can also be determined as follows:

(10) $$I=\int_{0}^{t}J(\tau )I_{g}(\tau ,t)\;d\tau ,$$

where *I*_g_(τ,*t*) is the growth current (at time *t*) of nuclei formed at time τ.

The η(*t*) function depends on the chosen technique for studying the electrochemical phase formation. In the case of variable overpotential, the currents associated with the processes of double-layer charging/discharging (*I*_c_) and a change in the concentration of adatoms (*I*_a_) must be taken into account in the current balance equation [36]:

(11) $$I=I_{c}+I_{a}+\underset{N}{\sum }I_{g},$$

(12) $$I_{c}=C_{d}s\frac{d\eta }{dt},$$

(13) $$I_{a}=zes\frac{d\Gamma }{dt},$$

(14) $$\Gamma =\Gamma_{0}\exp f\eta ,$$

where *C*_d_ is the specific capacity of the double electric layer, Γ is the concentration of single adatoms (monomers), and Γ_0_ is its initial value at *t* = 0.

In cyclic voltammetry, the time dependence of overpotential can be written as follows:

(15) $$\begin{array}{c} \eta =\nu t\;,\;0\leq t\leq t_{\lambda }\;(\mathrm{forward}\;\mathrm{scan}),\\ \eta =\nu (2t_{\lambda }-t)\;,\;t>t_{\lambda }\;(\mathrm{reverse}\;\mathrm{scan}), \end{array}$$

where ν is the scan rate, and *t*_λ_ is the reversal time. Then we get from Equations (12)–(15):

(16) $$\begin{array}{c} I_{c}+I_{a}=(C_{d}+ze\;f\Gamma_{0}\exp f\eta )\;s\nu \;,\;0\leq t\leq t_{\lambda },\\ I_{c}+I_{a}=-(C_{d}+ze\;f\Gamma_{0}\exp f\eta )\;s\nu \;,\;t>t_{\lambda }. \end{array}$$

The overpotential varies in a complex way under galvanostatic conditions [41,42,43], and η(*t*) can be obtained from Equations (11)–(14):

(17) $$\frac{d\eta }{dt}=\frac{i-\sum_{N}2\pi r^{2}i_{g}/s}{C_{d}+ze\;f\Gamma_{0}\exp f\eta },$$

where *i* is the applied cathode current density (*i* = *const*); the term $2\pi r^{2}i_{g}/s=0$ before the appearance of the first supercritical nucleus.

The numerical solution of systems of Equations (2), (7)–(9) (for the potentiostatic conditions), (2), (7)–(9), (15) and (16) (for the cyclic potential sweep), and (2), (7)–(9) and (17) (for the galvanostatic conditions) allows us to simulate the nucleation and growth processes in the listed cases. Calculations were performed using Microsoft Excel 2013. The introduction of nuclei was carried out gradually, when the integer value *N* was reached in accordance with Equation (2). The initial radius of each nucleus was $r_{0}=r_{с}(\eta )+\varepsilon$, where ε is the small quantity that made the nucleus supercritical. For calculations, the entire time scale (0–*t*) was divided into small time intervals ∆*t*_n_, the derivatives were replaced by finite differences, and the integrals were calculated via summation. The calculation parameters are specified in the following section.

## 3. Results and Discussion

### 3.1. Potentiostatic Electrodeposition

**Figure 1:**
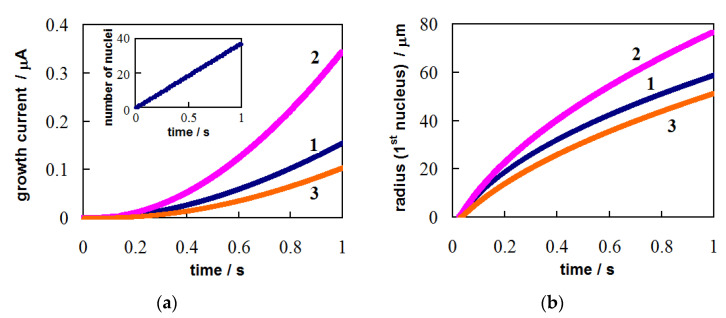
Calculated time dependences of (**a**) the total growth current and (**b**) the first nucleus radius under potentiostatic conditions (η = 40 mV). Inset: the time dependence of the number of nuclei. The values of bulk concentration of depositing ions (*c*_0_) and the exchange current density at the nucleus/electrolyte interface (*i*_0_): *c*_0_ = 1 × 10^19^ cm^−3^, *i*_0_ = 1 A cm^−2^ (curve 1, blue); *c*_0_ = 2 × 10^19^ cm^−3^, *i*_0_ = 1 A cm^−2^ (curve 2, pink); *c*_0_ = 1 × 10^19^ cm^−3^, *i*_0_ = 0.6 A cm^−2^ (curve 3, orange). Other parameters are provided in the text.

This is the simplest case since steady-state nucleation can be observed at η = *const* for some time at a stable concentration of adatoms and small coverage of the electrode with new-phase nuclei. Figure 1 demonstrates the time dependences of current (Figure 1a) and size of the first nucleus (Figure 1b) for these conditions. The *I*(*t*) and *r*_1_(*t*) dependences were calculated at *z* = 1, α = 0.5, σ = 7.5 × 10^−6^ J cm^−2^, *υ* = 1.7 × 10^−23^ cm^3^, *Т* = 300 K, *D* = 2 × 10^−5^ сm^2^ s^−1^, *K*_1_ = 10^7^ сm^−2^ s^−1^, *K*_2_ = 10^−2^ V^2^, *c*_0_ = 1 × 10^19^ cm^−3^ (curves 1 and 3) or *c*_0_ = 2 × 10^19^ cm^−3^ (curve 2), *i*_0_ = 1 A cm^−2^ (curves 1 and 2) or *i*_0_ = 0.6 A cm^−2^ (curve 3), and η = 40 mV. The above values are close to the parameters of silver electrodeposition on Pt from a nitrate solution [36,44]. The electrode surface area was taken equal to *s* = π(0.025)^2^ = 1.96 × 10^−3^ cm^2^ and ε = 10^−9^ cm. In this case, we neglected the current associated with the charging of the double electric layer (it appears on the experimental current transients as a sharp jump in current immediately after stepping the potential) and the nucleation time lag (it depends on many factors and varies within the relatively wide limits even for the same values of electrodeposition parameters [29]). Figure 1 shows that an increase in the bulk concentration of depositing ions (curve 2) leads to an increase in the size and growth rate of nuclei, and total growth current. The lower value of the exchange current density (curve 3) contributes to a decrease in the size of nuclei and the current.

Note that Equation (7) can be presented in the following form:

(18) $$i_{g}=\frac{1-\exp f(\eta_{p}-\eta )}{\frac{1}{i_{0}\exp\;\alpha f(\eta -\eta_{p})}+\frac{r}{zec_{0}D}}.$$

This formula can be useful for determining the contributions of the discharge and diffusion stages in each specific case by comparing the terms in the denominator. For example, the second term must exceed *i*_0_^−1^ by an order of magnitude to implement a pure diffusion regime. For the chosen calculation parameters, the effect of the discharge can be neglected only for large nuclei (*r* > 1.5 × 10^−4^ cm). Certainly, it should be taken into account that the higher the overpotential, the less *r*, at which the transition to purely diffusion control occurs. In molten salts, the *i*_0_ values are much higher, for example, the exchange current at the silver nucleus/nitrate melt interface can exceed 500 A cm^−2^ at 523 K [36,44]. This means that ceteris paribus, the growth process can be considered diffusion-controlled even for small silver nuclei with the size close to the critical one.

The results of our modeling do not contradict the experimental and theoretical results reported in [24,25,33,34]. We can also emphasize that our model describes correctly the limiting cases (diffusion or kinetic control), when one of the terms in the denominator of Equation (18) prevails. Therefore, there is no need to assume in advance a dominant growth mechanism. The very small nucleus growth is controlled by charge transfer; the transition to mixed control and then to diffusion control will be observed as the nucleus size increases. A similar conclusion was made in [33,34].

### 3.2. Cyclic Voltammetry

**Figure 2:**
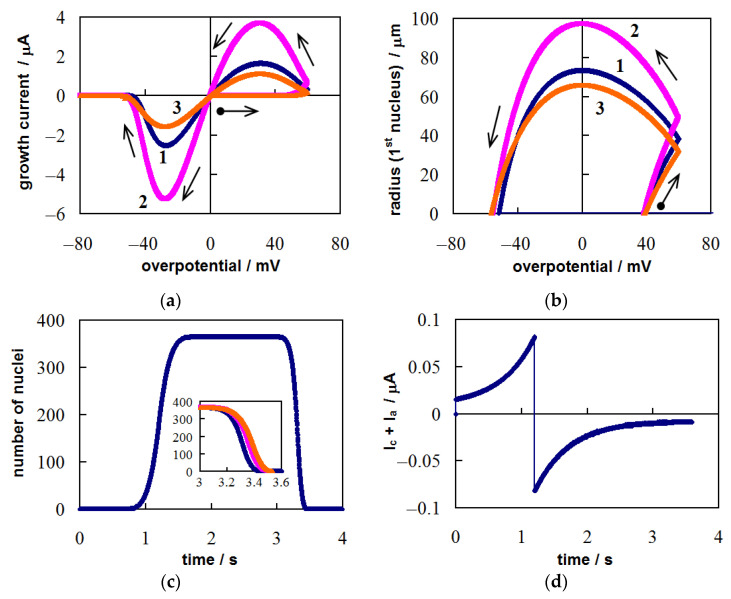
Calculated (**a**) CVs and (**b**) the overpotential dependences of the first nucleus radius. Calculated time dependences of (**c**) the number of nuclei and (**d**) the sum of the capacitive and adsorption currents. The dots indicate the moments of appearance of the first and last nuclei. Scan parameters: ν = 0.05 V s^−1^ and η_λ_ = 0.6 V. Values of *c*_0_ and *i*_0_: *c*_0_ = 1 × 10^19^ cm^−3^, *i*_0_ = 1 A cm^−2^ (curve 1, blue); *c*_0_ = 2 × 10^19^ cm^−3^, *i*_0_ = 1 A cm^−2^ (curve 2, pink); *c*_0_ = 1 × 10^19^ cm^−3^, *i*_0_ = 0.6 A cm^−2^ (curve 3, orange). Other parameters are indicated in the text.

In cyclic voltammetry, η(*t*) is described by Equation (15). Figure 2 presents the overpotential dependences of growth current (i.e., CVs) and size of the first nucleus, as well as time dependences of the number of nuclei and the sum of adsorption and capacitive currents under cyclic potential sweep conditions. These dependences were calculated at ν = 0.05 V s^−1^, η_λ_ = 0.6 V (*t*_λ_ = 1.2 s), *C*_d_ = 80 μF cm^−2^, Γ_0_ = 1.2 × 10^13^ cm^−2^, and the same values of *z*, α, υ, σ, *c*_0_, *i*_0_, *K*_1_, *K*_2_, *D*, *Т*, *s*, ε as in Section 3.1.

The shape of CVs (Figure 2a) is typical for the case of gradual formation and growth of non-interacting nuclei on an indifferent electrode [31,45,46]: a wide nucleation loop in the cathodic region (the current value on the reverse scan is higher than that on the forward scan at the same overpotential) and a stripping peak in the anodic region. The growth current begins to increase after the formation of the first supercritical nucleus (at η = 0.0383 V in this case). The overpotential decreases after the reversal point (η_λ_ = 0.6 V), but the growth of previously formed nuclei continues in the cathodic region. In addition, new nuclei appear and grow after η_λ_ (Figure 2c); in our case, the formation of the last 365th nucleus occurs at η = 0.0367 V. These processes lead to a significant increase in the current after η_λ_. The nuclei reach their maximum size in the crossover point at η = 0 (Figure 2b). In the anodic region, the sizes of the nuclei gradually decrease due to their dissolution. The growth current becomes zero after the largest (1st) nucleus dissolves. The sum of the capacitive and adsorption currents is shown in Figure 2d. If the contribution of these currents to the total current is significant, then it manifests itself on the CV as a deviation of the current from zero before the formation of the first nucleus and after the dissolution of all nuclei, as well as in the form of a crossed loop at the reversal point. Note that we analyzed the influence of the scan rate and the reverse potential in the case of nucleation with diffusion-controlled growth in [45]. In the case of mixed growth control, these factors have a similar effect on CV, i.e., peak currents decrease with increasing ν and increase with increasing η_λ_.

The growth current and the size of the nuclei increase as the concentration of the depositing ions increases (curve 2). The decrease in the exchange current density has the opposite effect (curve 3). Both of these factors lead to a slight increase in the dissolution time of the nuclei (inset in Figure 2c). As in the case of diffusion-controlled growth [45], an increase in ν and a decrease in η_λ_ causes a decrease in the number and size of nuclei.

Note that in practice, narrow loops with a weakly pronounced maximum are usually recorded. This may be due to various reasons, including the nucleation in a narrow time interval compared to the time scale of the experiment almost immediately after the beginning of the potential sweep in the cathodic direction or a mutual influence of nuclei. For the diffusion-controlled growth of a single Ag nanocluster on a nanoelectrode, the results of calculations using the similar approach [32] agree both qualitatively and quantitatively (in the cathode part) with the experiment [29].

### 3.3. Galvanostatic Electrodeposition

**Figure 3:**
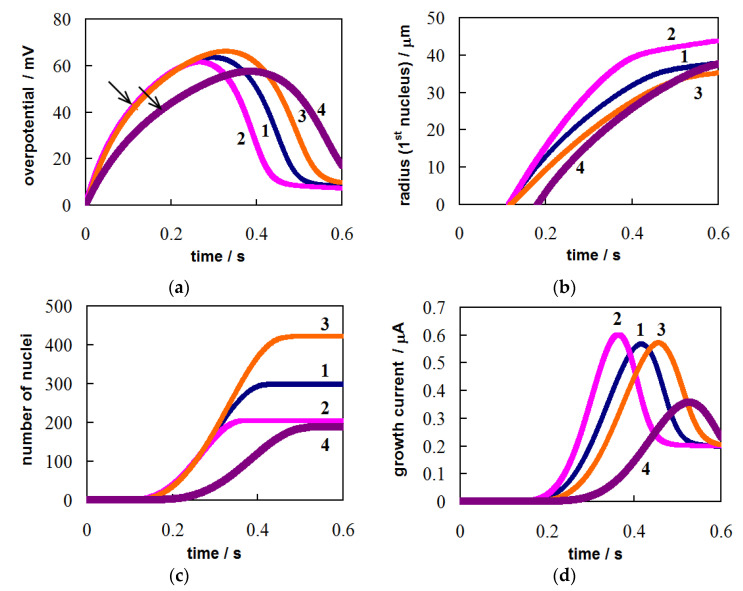
Calculated time dependences of (**a**) the overpotential, (**b**) the first nucleus radius, (**c**) the number of nuclei, and (**d**) the total growth current. Applied current density: *i* = 10^−4^ A сm^−2^ (curves 1–3) and *i* = 6 × 10^−5^ A сm^−2^ (curve 4). Values of *c*_0_ and *i*_0_: *c*_0_ = 1 × 10^19^ cm^−3^, *i*_0_ = 1 A cm^−2^ (curve 1, blue); *c*_0_ = 2 × 10^19^ cm^−3^, *i*_0_ = 1 A cm^−2^ (curve 2, pink); *c*_0_ = 1 × 10^19^ cm^−3^, *i*_0_ = 0.6 A cm^−2^ (curve 3, orange). Other parameters are indicated in the text.

The analysis of multiple nucleation/growth processes is difficult under galvanostatic conditions even for independent nuclei due to the impact of many factors, including complex η(*t*) dependence, charge/discharge of the double electric layer, changes in the concentration of adatoms, changes in mass transfer conditions, the mutual influence of nucleation rate and growth rate of nuclei [24,35,36,37]. The calculated dependences of η(*t*), *r*_1_(*t*), *N*(*t*) and Σ*I*_g_(*t*) are presented in Figure 3. In the calculations, we used the initial conditions Γ(0) = Γ_0_, η(0) = 0, *N*(0) = 0, *r*(0) = 0, the same values of *z*, α, υ, σ, *c*_0_, *i*_0_, *K*_1_, *K*_2_, *D*, *Т*, *C*_d_, Γ_0_, *s*, ε as in Section 3.1, and *i* = 10^−4^ A сm^−2^ (curves 1–3) or *i* = 6 × 10^−5^ A сm^−2^ (curve 4).

Switching on the cathodic current leads to the charging of the double layer and an increase in the concentration of adatoms and overpotential (Figure 3a). The first supercritical nucleus appears at η = 0.0425 V (curves 1–3) or η = 0.0411 V (curve 4). At this point, the concentration of adatoms is 6.20 × 10^13^ (or 5.88 × 10^13^) cm^−2^, i.e., ~3.5% from the Ag monolayer. The progressive formation of nuclei and an increase in their size (Figure 3b,c) somewhat slows down the growth of η(*t*). The overpotential continues to increase until the total growth current of all nuclei becomes equal to the applied current, Σ*I*_g_ = *is*. This moment corresponds to the maximum overpotential. The overpotential decreases, when Σ*I*_g_ > *is*. For this reason, the double layer is discharged and Γ is reduced; the prevailing ion flux from the electrode surface into the electrolyte bulk arises. At the same time, the total growth current continues to increase for some time (Figure 3c,d) due to the appearance of new nuclei up to η ≈ 0.042 V. After the termination of the nucleation process, Σ*I*_g_ gradually decreases to the *is* value. At the final stages, a slow growth of nuclei is only observed at the almost constant low overpotential (Figure 3b).

Under galvanostatic conditions in contrast to the two previous cases, the number of nuclei decreases as the concentration of depositing ions increases (see Figure 3c). At the same time, nuclei are larger at higher concentrations (see Figure 3b) due to increased growth currents. Therefore, the overpotential begins to decrease earlier (see Figure 3a) and the nucleation period is reduced, which leads to a decrease in *N*. A lower exchange current density at the nucleus/electrolyte interface promotes an increase in *N* due to a prolongation of the nucleation period; the size of the nuclei decreases. A decrease in the applied current density leads to a decrease in the maximum overpotential and *N*. Similar regularities were found in experimental galvanostatic studies of the formation and growth of silver nano- and microcrystals in nitrate melts [44,47].

### 3.4. Example of Using the Model

We considered the experimental CV obtained in the study of the formation and growth of a single nanosized silver nucleus on a 100 nm-radius Pt electrode from the solution containing 100 μMAg_2_SO_4_ and 0.1 M H_2_SO_4_ in [29].

Let us first assume that the growth of the nucleus is diffusion controlled. Then Equation (18) is transformed into

(19) $$i_{g}=zec_{0}D[1-\exp f(\eta_{p}-\eta )]/r,$$

**Figure 4:**
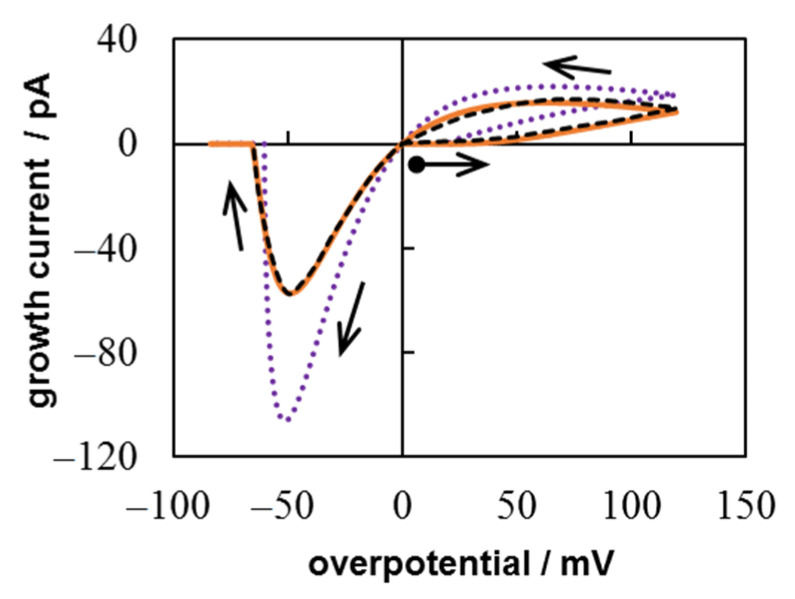
Comparison of simulation results for diffusion growth control at *D* = 1.5 × 10^−5^ сm^2^ s^−1^ (dotted line) and mixed growth control at *D* = 1.72 × 10^−5^ сm^2^ s^−1^, *i*_0_ = 6.32 × 10^−2^ A cm^−2^ and α = 0.128 (solid line) with experimental CV (dashed line) [29].

Since we can neglect the first term in the denominator of Equation (18) in the case diffusion-controlled growth. The result of the numerical calculation of the system including Equations (3), (5), (8), (15), and (19) in comparison with the experimental CV is shown in Figure 4. The simulation was performed at *c*_0_ = 1.2 × 10^17^ cm^−3^, *Т* = 300 K, *D* = 1.5 × 10^−5^ сm^2^ s^−1^, ν = 0.05 V s^−1^, ∆*t* = 6.5 × 10^−4^ s, *t*_λ_ = 2.4 s (η_λ_ = 0.12 V), *t*_0_ = 0.4 s (η_0_ = 0.02 V), where *t*_0_ and η_0_ are the time and overpotential of the supercritical nucleus formation, respectively. The diffusion coefficient *D* = 1.5 × 10^−5^ сm^2^ s^−1^ was found during chronoamperometric data treatment in [29] under the assumption of purely diffusion growth control. Significant discrepancies are clearly visible both in the cathode and in the anodic region (dotted and dashed lines).

Now let us make a nonlinear fitting by the Levenberg–Marquardt algorithm to determine *D*, *i*_0_, and α using formula (7) (or (18)) instead of Equation (19), i.e., considering mixed growth control. In this way, we can achieve good agreement between the experimental and simulated curves (solid and dashed lines in Figure 4). The best agreement was obtained at *D* = 1.72 × 10^−5^ сm^2^ s^−1^, *i*_0_ = 6.32 × 10^−2^ A cm^−2^, and α = 0.128. Thus, the proposed model can provide a more accurate interpretation of the experimental results.

**Figure 5:**
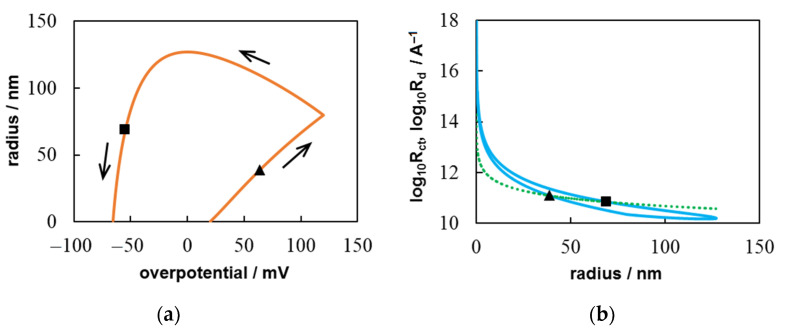
Calculated dependences of (**a**) the nucleus radius against overpotential and (**b**) the decimal logarithms of the contributions of the charge transfer (*R*_ct_, solid line) and diffusion (*R*_d_, dotted line) limitations against the nucleus radius. The symbols indicate the points, at which *R*_ct_ = *R*_d_.

In addition, the calculation allows tracing the change in the size and growth regime of the nucleus during the change in the overpotential (Figure 5). The maximum calculated nucleus radius is about 126 nm. This does not contradict the AFM observations [29], which show that the nucleus can slightly extend beyond the Pt nanoelectrode. Simulations demonstrate that the charge transfer controls the growth of small nucleus. For this CV, the contribution of kinetic limitations ($R_{\mathrm{ct}}={\left[2\pi r^{2}i_{0}\exp\;\alpha f(\eta -\eta_{p})\right]}^{-1}$) is an order of magnitude greater than the contribution of diffusion limitations ($R_{d}={\left[2\pi rzec_{0}D\right]}^{-1}$) up to *r* < 5 nm. The *R*_ct_ and *R*_d_ values will become equal at η ≈ 64 mV (triangle in Figure 5). Further, the diffusion contribution will prevail until the transition to the anode region. As the nucleus dissolves, the effect of diffusion limitations will gradually decrease, and the discharge limitations will increase.

## 4. Conclusions

Within the framework of the general model, the processes of formation and mixed-controlled growth of independent new-phase nuclei on an indifferent electrode under constant and variable overpotential are analyzed. The influence of various factors on the limiting stage of growth is discussed. Simulation results for potentiostatic electrodeposition, galvanostatic electrodeposition, and cyclic voltammetry are presented. The initial sections of potentiostatic current transients, complete cyclic voltammograms, and galvanostatic overpotential transients, as well as time dependences of the number of nuclei, their sizes, and growth currents are calculated. The effect of the bulk concentration of depositing ions (*c*_0_) and the exchange current density at the electrolyte/nucleus interface (*i*_0_) on these dependences is analyzed. A significant difference between galvanostatic electrodeposition and other investigated cases was demonstrated: an increase in *c*_0_ and *i*_0_ leads to a decrease in the number of nuclei due to a faster decrease in the overpotential and the nucleation period reduction. The size and growth rate of nuclei decrease with decreasing *c*_0_ and *i*_0_ in all cases. Thus, this model can be used to select the optimal electrodeposition conditions by determining the influence of various experimental factors (concentration of deposited ions, temperature, etc.) on the number and size of new-phase nuclei. In addition, the analysis of the experimental dependences current vs. time, current vs. overpotential, potential vs. time using this model is useful for elicitation of the electrocrystallization parameters, including the diffusion coefficient, exchange current density, transfer coefficients, etc.

## Data Availability

The data presented in this study are available on request from the corresponding author.
